# ECG classification using 1-D convolutional deep residual neural network

**DOI:** 10.1371/journal.pone.0284791

**Published:** 2023-04-25

**Authors:** Fahad Khan, Xiaojun Yu, Zhaohui Yuan, Atiq ur Rehman

**Affiliations:** 1 School of Automation, Northwestern Polytechnical University, Xi’an, China; 2 Department of Electrical and Computer Engineering, COMSATS University Islamabad, Abbottabad Campus, Pakistan; 3 Artificial Intelligence and Intelligent Systems Research Group, School of Innovation, Design and Engineering, Mälardalen University, Västerås, Sweden; 4 Department of Electrical and Computer Engineering, Pak-Austria Fachhochschule Institute of Applied Sciences and Technology, Haripur, Pakistan; Menoufia University, EGYPT

## Abstract

An electrocardiograph (ECG) is widely used in diagnosis and prediction of cardiovascular diseases (CVDs). The traditional ECG classification methods have complex signal processing phases that leads to expensive designs. This paper provides a deep learning (DL) based system that employs the convolutional neural networks (CNNs) for classification of ECG signals present in PhysioNet MIT-BIH Arrhythmia database. The proposed system implements 1-D convolutional deep residual neural network (ResNet) model that performs feature extraction by directly using the input heartbeats. We have used synthetic minority oversampling technique (SMOTE) that process class-imbalance problem in the training dataset and effectively classifies the five heartbeat types in the test dataset. The classifier’s performance is evaluated with ten-fold cross validation (CV) using accuracy, precision, sensitivity, F1-score, and kappa. We have obtained an average accuracy of 98.63%, precision of 92.86%, sensitivity of 92.41%, and specificity of 99.06%. The average F1-score and Kappa obtained were 92.63% and 95.5% respectively. The study shows that proposed ResNet performs well with deep layers compared to other 1-D CNNs.

## Introduction

Cardiovascular diseases (CVDs) are one of the major threats faced by humans [1, 2]. Normal heartbeat depends on factors such as age, body size, diet, emotions, and activity. Too fast or slow heartbeat conditions is medically known as palpitations. The rhythmic irregularities of heart are commonly known as arrhythmias or Cardiac Dysrhythmia. Arrhythmia is broadly categorized into two types: non-life-threatening and life-threatening.

ECG is a very easy, non-invasive, highly efficient, and useful tool to monitor and identify arrhythmia by measuring the electrical activity of heart [3]. In arrhythmias, there are three main malfunctions of heart; heart beats become slow, i.e, below 60 bpm (Bradycardia), fast over 100 bpm (Tachycardia), or irregular (Fibrillation), such that proper proportional of blood to body parts cannot be maintained by heart. The precise and early-stage detection and classification of ECG signals is critically essential for patient acute heart conditions and treatment [4]. To obtain the proper ECG record doctors use Holter and loop recorder to the suspected arrhythmias for a minimum duration of 24 hours. Later on, the ECG record is analyzed using computer programs for detecting specific type of arrhythmia, which is time-consuming procedure. Although arrhythmias have two types; non-life-threatening and life-threatening, however, arrhythmias are mostly detrimental. The type of arrhythmia is determined by the ECG signal shape and other morphological factors.

The Association for the Advancement of Medical Instrumentation (AAMI) has categorized five types of the non-life-threatening arrhythmia signals: non-ectopic beat (N), supra ventricular ectopic beat (SVEB or S), ventricular ectopic beat (VEB or V), fusion (F), and unknown beat (Q). The typical ECG waveform constitutes of the primary wave groups, such as P wave, QRS wave group, and T wave, which are all represented in a single ECG period. The energy and physiological implications of each waveform information and distinctive wavelet is different. The QRS wave group has more energy and amplitude than the P and T wave groups.

Feature extraction and pattern classification are employed in the classification of signals or images. The easiest way of ECG feature extraction can be obtained by extracting sampled points from an ECG raw signal. However, the extracted version has large features that severely affects the classifier efficiency. Other methods used for feature extraction is the morphological and/or statistical techniques. Another method for features extraction from raw signals is the morphological and/or statistical techniques. An example of such technique is the one where the RR interval measurement that uses time between the R peaks of two heartbeats, is used for features. Another statistical method for obtaining ECG features is Independent Component Analysis (ICA). In [5], training of an ECG classifier is conducted by morphological and statistical features.

The literature presents numerous techniques for the detection of heartbeat diseases including threshold-based methods [6], wavelet transform (WT) [7, 8], digital filter-based methods [9], morphology-based methods [10], non-invasive methods [11], and so on. Feature extraction of ECG signal using WT includes discrete cosine transform (DCT), continuous wavelet transform (CWT), and discrete wavelet transform (DWT). For instance, Khorrami et al. extracted features in ECG classification using DCT coefficients [12]. Furthermore, they applied CWT and DWT to obtain features for ECG classification, and provided performance classification among DCT, CWT, and DWT. In [13], the wavelet packet decomposition (WPD) is used to obtain ECG features and classification was calculated using wavelet packet entropy (WPE) and random forests (RF).

Machine learning (ML), which is a subset of artificial intelligence is being utilized for the detection and analysis of different diseases. The diagnostic tools are used in ML for the examination of diseases in human body [14, 15]. Deep learning (DL) is a subset of ML, have been widely used for the diagnostic of ECG signals and other diseases. In bio-informatics, DL techniques are extensively utilized due to their remarkable performance [14]. DL models utilize deep neural networks (DNNs), which are further categorized into CNNs, long-term short-term memory (LSTM), and recursive neural networks (RNNs). Among these, CNNs are extensively applied in various fields. The CNNs [16] and DNNs [17] have been successfully applied in classification of ECG signals.

In radiological image analysis, DL approaches are devised that provides outstanding performance [18, 19]. Recently, CNNs have shown greatest research potential since they are suitable for multi-dimensional inputs, such as ECG time series data (1-D), and images (2-D and 3-D) input [20]. Owing to the wide utilization of CNN in diverse applications [21, 22], CNN have proved effective in classification of ECG signals [23, 24]. CNN have been implemented for ECG classification that provides better accuracy and performs feature extraction by directly using input heartbeats [25, 26].

In [27], RNN is investigated where the training process was performed using the feature extraction obtaining an average accuracy of 98.06% for the classification of four different types of arrhythmias. The classification and feature extraction of 1-D ECG is performed in [26], where an adaptive CNN model is used enabling a classification accuracy of 96.72%. Moreover, their CNN model is generic due to its parameter invariance making it applicable to any real ECG dataset. Authors in [28] proposed an ECG classification model that provides a Chi2 selector, homeomorphically irreducible tree (HIT) pattern feature generator, and SVM classifier. Their model provides an accuracy of 92.95% and 97.18% classifcation accuracy for seven- and four-class ECG. A novel transformer-based DL model is presented in [29] that performed remarkable for MIT-BIH arrhythmia and MIT-BIH atrial fibrillation databases. In [30], an ECG classification model is investigated that employs a bidirectional long short-term memory networks (Bi-LSTMs) and a generative adversarial network (GAN). As reported therein, their model achieved an overall accuracy of 98.7%. On a sizable ECG dataset with more than 10,000 12-lead ECGs, it achieved accuracy scores for seven- and four-class arrhythmia classification of 92.95% and 97.18%, respectively. The accuracy of the model is on level with a DL model.

A deeper 34-layer 1-D CNN model was proposed for the classification of twelve arrhythmia types present in the time-series and obtained an average accuracy of 97.03% [31]. Li presented a 1-D CNN with five layers in additional to input and output layers for the classification of the five typical types of arrhythmias, i.e., normal, left bundle branch block, right bundle branch block, atrial premature contraction and ventricular premature contraction, achieving an accuracy of 97.5% [32]. A nine-layer 2-D CNN model was applied for an automatic classification of five different heartbeat arrhythmia types achieving an accuracy of 94.03% and 93.47% in the arrhythmia classification in original and de-noising heartbeats respectively [33]. An ECG monitoring system integrated with the Impulse Radio Ultra Wideband (IR-UWB) radar using CNN is provided with an accuracy of 88.89% [34].

Motivated by the above developments conducted in ECG classification, we have developed an ECG classification mechanism using 1-D convolutional for deep layers ResNet. Following are the main contributions of this study:

- Development of a model that does not require a separate feature extraction procedure, rather employs convolutional and pooling layers in succession for robust features extraction from the input ECG signals, therefore, the preprocessed ECG signals are trained and classified directly.
- Integration of SMOTE that generates synthetic minority data samples using the k-nearest neighbor technique enabling an equal number of samples for all the five heartbeat classes that are used in appropriate training of the ResNet model.
- Designing a deeper ResNet model for ECG classification that performs significantly well with an increase in depth of the network providing a high classification accuracy for the real testing ECG dataset.

The rest of paper is organized such that Section II provides material and methods. In Section III the proposed ResNet model is presented. Experimental setup and results are discussed in Section IV and conclusions are highlighted in Section V.

## Materials and Methods

**Fig 1:**
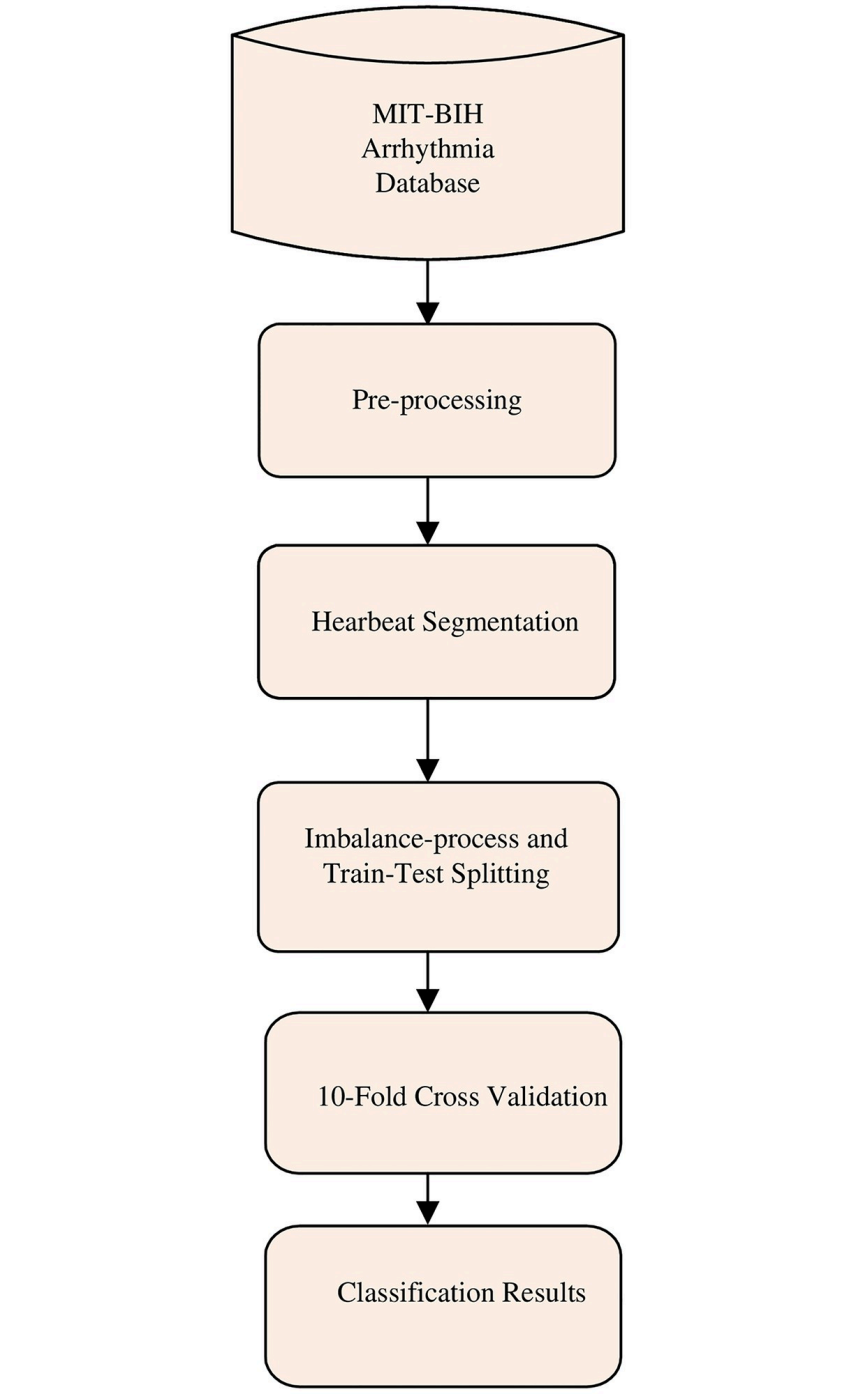
Main procedure involved in the classification of ECG.

The main procedure involved in the classification of ECG is provided in Fig 1.

### MIT-BIH dataset

The MIT-BIH databases comprises of numerous sub-databases that record specific types of ECG. We have utilized PhysioNet MIT-BIH Arrhythmia [35], which is freely available and widely utilized heartbeat dataset for performance evaluation of numerous ECG categorization algorithms. This standard database comprises of a total of 48 two channels ECG signal recordings taken from 47 individuals under observation. The length and sampling rate of the recorded data are thirty minutes and 360 Hz respectively.

MIT-BIH ECG dataset is a collection and processed data of heartbeat signal, which were marked and manually interpreted by experts into 15 arrhythmia classes. However, AAMI Standard grouped these 15 arrhythmia classes into five types (one normal and four with arrhythmia), which are described in Table 1.

**Table 1:** MIT-BIH verses AAMI 5 heartbeat classes grouping.

AAMI 5 heartbeats	MIT-BIH 15 heartbeats
Normal (N)	Normal beat Left bundle branch block beat (LBB) Right bundle branch block beat (RBB) Atrial escape Nodal (junctional) escape
Ventricular ectopic (V)	Premature ventricular contraction beat (PVC) Ventricular escape
Supraventricular ectopic (S)	Atrial premature contraction beat (APC) Supraventricular premature beat Aberrated atrial premature beat Nodal (junctional) premature beat
Fusion (F) Unknown (Q)	Fusion of non-ectopic and ventricular beat Paced beat Fusion of paced and normal beat Unclassifiable beat

### ECG data preprocessing

The preprocessing of ECG signal is very useful in improving the efficiency of the dataset and enables the extraction of different heartbeats from a particular ECG waveform. The preprocessing stage usually involves the steps of noise removal from ECG waveform and peak detection for segmentation of ECG signal into different heartbeat classes.

In this study, the single heartbeats from the continuous ECG were obtained using the Pam-Tompkins algorithm. Since QRS complex is the most prominent portion in the ECG, it serves as the foundation for practically all computerized ECG diagnostic methods. Here detecting every R peak is the same as obtaining a single heartbeat. The Pam-Tompkins algorithm uses a number of steps in finding R-peaks in the ECG signal, including derivative, squaring, integration, edge recognition, and search approaches for R-peaks. Finally, after detecting the QRS waveform and obtaining the P, R, and T peaks, segmentation of the single heartbeat is completed. The majority of pathological information is contained in each heartbeat, which is used for disease finding. In this paper, there are a total of 109446 heartbeats with a sampling frequency of 125 Hz taken from 44 records, which are utilized in the training and testing analysis of the proposed 1-D convolution neural network model.

### Data imbalance process using SMOTE

**Fig 2:**
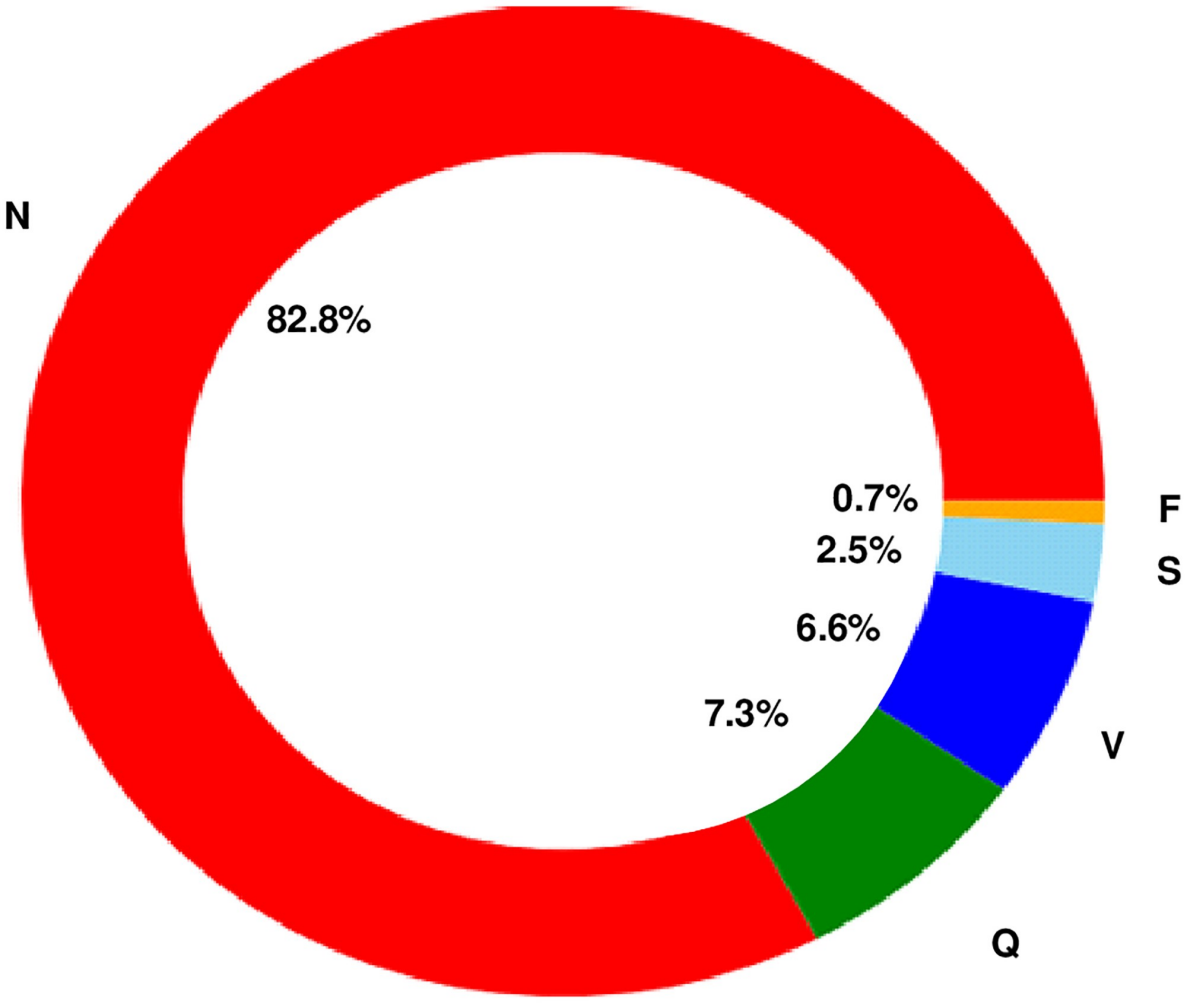
Distribution of heartbeats in different classes of the MIT-BIH ECG database.

The distribution of heartbeats in the different classes of the MIT-BIH ECG database is not uniform as shown in Fig 2. According to ANSI-AAMI, approximately 80% of heartbeats belong to N class, while the remaining 20% heartbeats are from V, S, F, and Q classes. Since, the heartbeat samples in N class is far higher than the samples in minority class, therefore, MIT-BIH classes presents a highly imbalance heartbeat dataset. Such class-imbalanced dataset results in misclassification due to the biased decision in favor of the majority class. There are different methods to solve the issue of class imbalance in datasets that employs balancing at algorithm level, data level, cost-sensitive methods, and integration methods [36]. Data-level approaches are extensively applied due to their advantages of being algorithm-independent and simplified operations. Its core concept is re-sampling, which includes both oversampling and under-sampling. Random oversampling (ROS) and random under-sampling (RUS) are the most basic re-sampling techniques. Other re-sampling techniques are EasyEnsemble [37], KNNOR [38], and SMOTE [39].

SMOTE is a famous technique that can remove class-imbalance problem in dataset. In traditional oversampling process, the minority data is merely duplicated from the minority dataset. Although dataset samples are increased, the oversampling does not provides any additional knowledge or variation to the classification model.

SMOTE generates synthetic minority data samples using the k-nearest neighbor technique. SMOTE begins by selecting random data from the minority class, after which the k-nearest neighbors of data are determined. SMOTE synthetic data *x*^*syn*^ are generated by the following mathematical relation:

$$\begin{array}{c} x^{syn}=x^{i}+\left(x^{j}-x^{i}\right)\delta \end{array}$$ (1)

Where *x*^*i*^ is the instance of minority class (sample) under consideration, *x*^*j*^ is the K-nearest neighbors of *x*^*i*^, *δ* is a vector with elements having random values from [0, 1]. Therefore, there are two steps to generate synthetic data samples in SMOTE:

- Firstly, obtain the difference between the minority class (sample) under consideration and its nearest neighbor. The obtained difference is multiplied by a vector *δ*.
- The calculated value is added to the minority class (sample) under consideration to generate *x*^*syn*^ along the line between vectors *x*^*i*^ and *x*^*j*^.

Table 2. provides class wise heartbeats before and after using SMOTE on training dataset. SMOTE creates data balancing where all the heartbeat classes have an equal number of samples in the training dataset. It is important to note that except majority class (N) all the other four classes (S, V, F and Q) are oversampled using SMOTE.

**Table 2:** Total heartbeats in training dataset classes before and after SMOTE.

Heartbeat class	Samples in actual dataset	80% Training samples	20% Testing samples	SMOTE samples
*N*	90589	72471	18118	72471
*Q*	8039	6431	1608	72471
*V*	7236	5788	1448	72471
*S*	2779	2223	556	72471
*F*	803	641	162	72471
*Total*	109446	87554	21892	362355

### Convolution neural network and residual neural networks

CNN are capable of extracting most appropriate features from input data by using convolution operation. Since ECG and electroencephalogram (EEG) are time-series signals, therefore, 1-D convolution is applied to process these signal.

In CNN, the input of each layer is obtained from the output of the preceding layer [40]. The fundamental unit of CNN is comprised of input layer, convolution layer, activation function and output layer. For the input x, the overall operations involved in CNN can be expressed as:

$$\begin{array}{c} y=f(Wx+b) \end{array}$$ (2)

where *y* represents the output, *f* denotes the ReLU function, *W* is the convolution matrix, and *b* is the bias.

**Fig 3:**
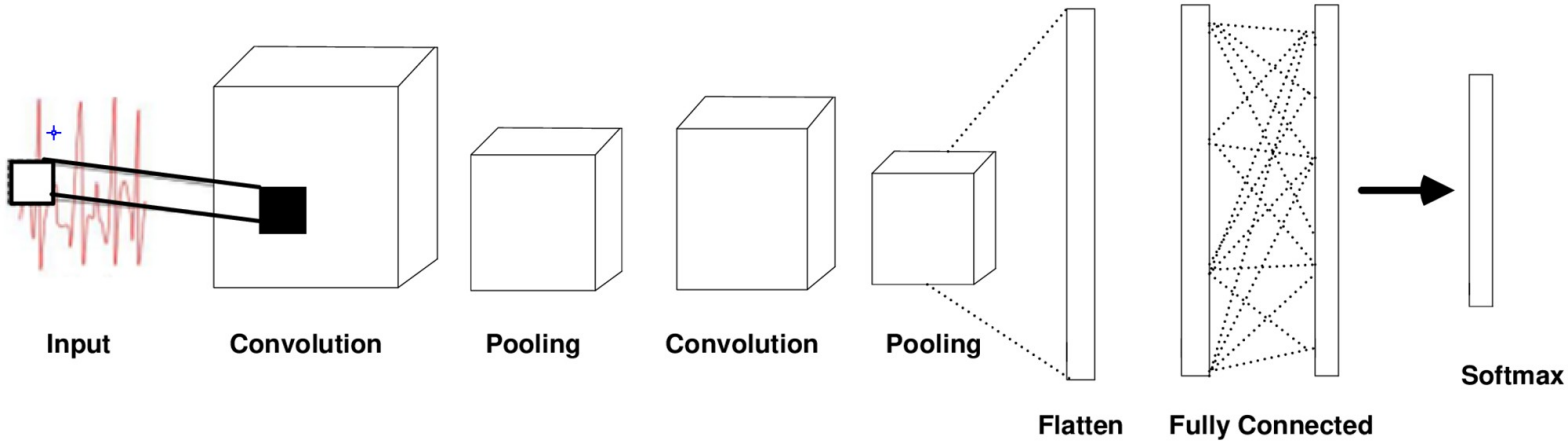
CNN with sequence of layers from input to output.

In complex CNN architectures, there are numerous other layers incorporated in basic structure of CNN. These layers consist of multiple convolutional layers, pooling (down sampling) layers, flatten layer, fully connected (FC) layer, and finally an output layer. CNN with sequence of layers from input to output is provided in Fig 3. A brief description of CNN is provided here:

1. Input Layer-It is the first layer of CNN containing input data that may be time-series (1-D) or images (2-D or 3-D).
2. Convolutional layers-These layers are responsible for convolutional operations on input dataset to extract significant features.
3. Convolution layer employ kernels (filters) that has weight and a bias. The kernel has a matrix of weights multiplied with input data to obtain features. For time series input data, 1-D convolution is used, while image have 2-D and 3-D convolutions.
4. Activation function-In a CNN model, each convolution layer is usually followed by an activation function. The Rectified Linear Unit (ReLu) function is a popular choice for most CNN models.
5. Pooling layers-Because the output of a convolutional layer contains redundancy, extracting relevant features from an input data is difficult. The pooling layer reduces the number of parameters by repeatedly extracting feature value from a group of cells by some pooling method. There are a variety of pooling methods available for use in different pooling layers. Tree pooling, gated pooling, average pooling, minimum pooling, maximum pooling, global average pooling (GAP), and global maximum pooling are among some of the techniques available. The most popular pooling methods are max, min, and GAP. The over-fitting and extensive computations are reduced after processing through the pooling layer.
6. Dropout-The term “dropout” refers to the process of removing units (both hidden and visible) from a neural network to considerably prevent the over-fitting of the underlying model.
7. Flatten and FC layers-The flatten layers are placed before the output layer and they perform the conversion of multidimensional output into a vector. Flatten layer output is used as input for the FC layers. FC layers use the extracted features obtained from pooling layer to perform early classification on input data. Basically, the output matrix from the pooling layer is flattened to a one-dimensional vector and used as input for the FC layers.
8. Softmax/ logistic layer-It is connected at the end of FC layers and is used to finally classify the training data into classes. For binary classification problem, logistic is used with sigmoid activation function whereas softmax is for multi-classification.

**Fig 4:**
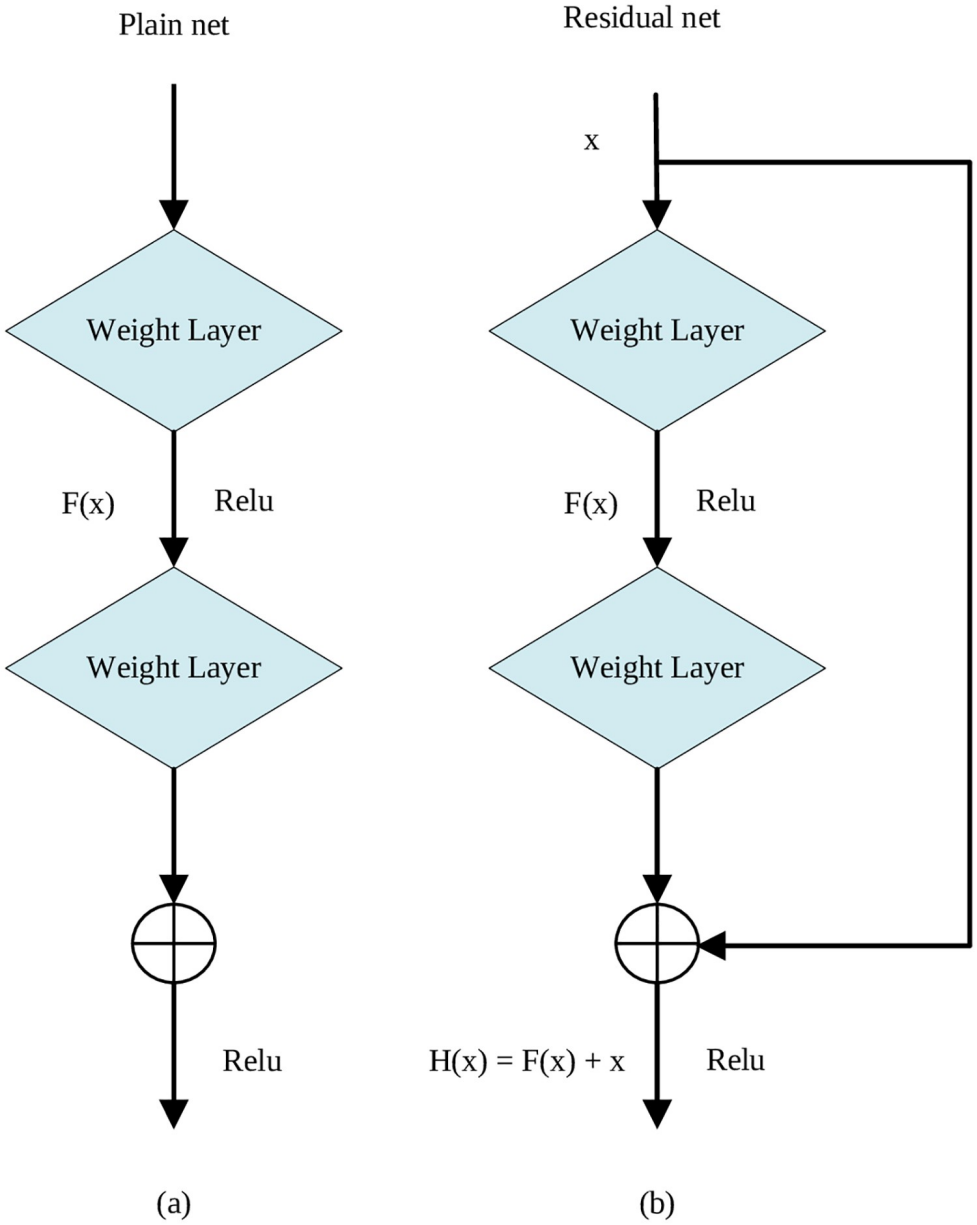
(a) Plain network and (b) A residual network.

The structure of a plain CNN is shown in Fig 4(a). The filtering process occurs whenever the data goes from the convolution or pooling operations in previous layers. The overall objective of the processing on data is the size reduction of input vector. In general, it is desirable to decrease network parameters so that the problem of over-fitting does not happen. Although the learning potential of neural network increases with deepening the network, however, increasing number of layers may cause gradient dissipation or gradient explosion, which will degrade the performance and affects convergence [41]. To overcome gradient vanishing explosion in deeper networks, we employ residual networks (ResNets). ResNets avoids the gradient dissipation or gradient explo-sion issues in deep layer networks, thereby enabling improved accuracy and optimised performance.

The ResNet building block is shown in Fig 4(b). The ResNet has input parameter *x* and output target *H*(*x*) with a short circuit or skip connection structure. These shortcut connections in ResNet directly learn the residual given by the formula:

$$\begin{array}{c} F(x)=H(x)-x \end{array}$$ (3)

The targeted output *H*(*x*), therefore, becomes:

$$\begin{array}{c} F(x)=H(x)+x \end{array}$$ (4)

In plain network structure, the processing of each layer comes from the output of the previous layer. In ResNet, input data do not merely depends on previous layer output but preceding network structure. As a consequence, sufficient information is extracted from input features [40]. The shortcut connections by-passes two or more layers and directly perform identity mapping. Therefore, such networks avoid performance degradation and accuracy reduction issues faced in plain networks due to large convolution layers.

## Proposed resnet model

In DL, CNN has attracted popularity in recent years due to its outstanding performance for image and speech recognition applications. In CNN, feature learning is achieved by extracting useful local features from input data automatically.

**Fig 5:**
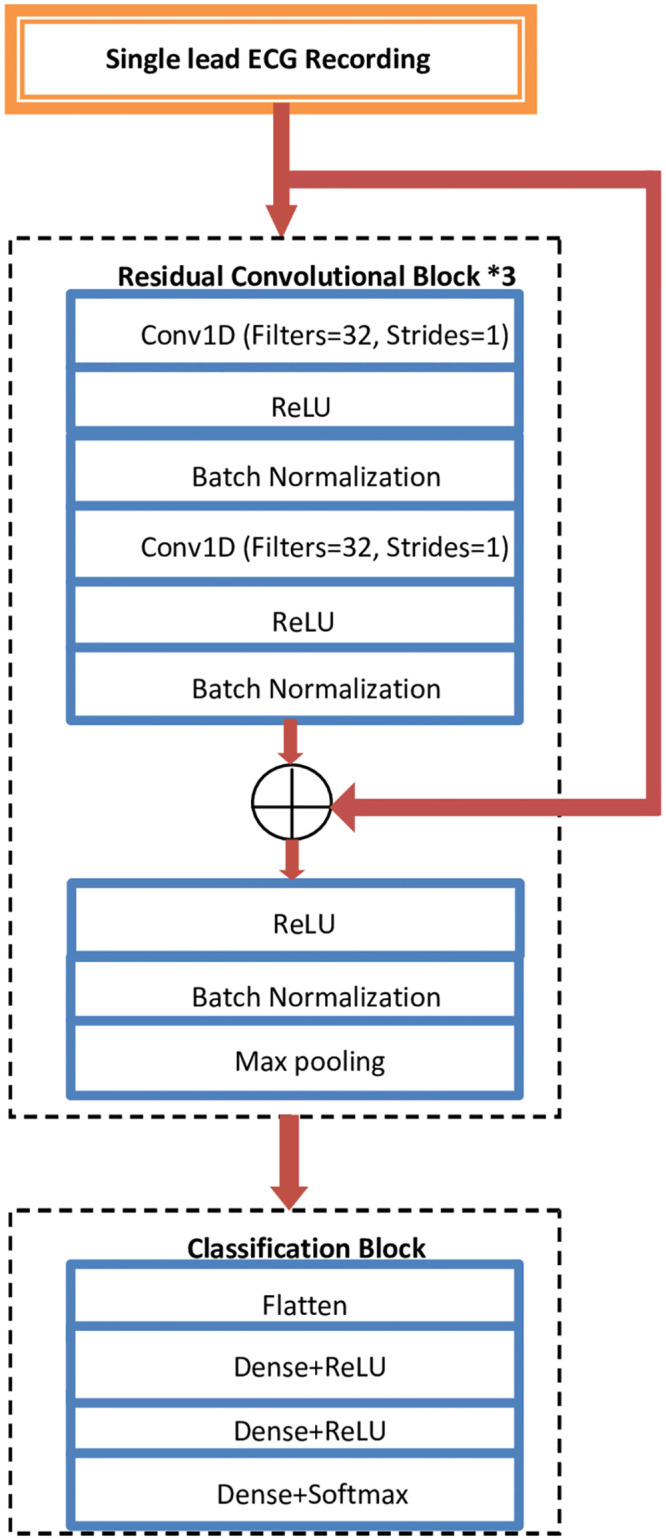
The architecture of the proposed ResNet model.

Fig 5 provides the architecture of the proposed deep ResNet model. The proposed model contains deep layer architecture with three residual convolution blocks preceding a classification block. The input layer has 1-D ECG data with 188 samples. The number of channel is one because the ECG data is taken from single lead. The proposed model has six convolutional layers and three max pooling layers providing robust features extraction from the input ECG signals. The proposed model layer-wise architecture is explained as follows.

The ECG data is passed to down-sampling block, which consists of 1-D convolution and BN layer, and a ReLU activation function. The convolution layer has 64 filters, a kernel of 3, and stride of 2. The first residual block constitutes of series of two sets of convolutional layer with ReLU applied as an activation function and BN. The skip circuit follows this and then a ReLU activation function is used to reduce over-fitting. After that, BN is used to accelerate CNN training process by reducing internal covariate shift. The maximum-pooling (also known as max-pool) is added, which computes the maximum value in each patch of the feature map and enables to diminish the size of the feature. Maxpool1D with pool size of 5 and strides of 2 is used that performs max-pooling operation on spatial domain signal.

The second and third residual blocks have the same structure as the first residual block, i.e, Convolutional layer-ReLU-BN that are added to the output of down-sampling block through a skip connection, thereafter, ReLU, BN, and max pooling is performed.

Finally, the classification stage has a flatten layer that aims to translate the multi-dimensional information into 1-D information. Following flatten layer, there are 2 full connection dense layers with ReLU function and one dense layer with softmax for five heartbeats classification.

The proposed ResNet model has advantage over the plain network such that the input data does not merely depends on previous layer output but preceding network structure. As a result, sufficient useful information is extracted from input features of ECG. Moreover, the shortcut connections in the ResNet architecture by-passes two or more layers and directly performs identity mapping. Therefore, proposed network avoids performance degradation and accuracy reduction issues faced in plain networks due to large convolution layers.

## Experimental setup and results

### Training and testing

In this work, the real dataset is split into training and testing portions with 80:20 train-test split. 10-fold cross validation was used during the training process. Inside the training data, we further split into 80% to be the actual training data and 20% as a validation data. The validation data enables the monitoring of training process and prevent the model from over-fitting. Due to imbalance nature of heartbeat classes in original data, the real training data was oversampled using the SMOTE. This data was provided as an input to proposed deep ResNet model. After training the model, the testing procedure is conducted by using 20% of the real test dataset that was not oversampled by any method. The testing data is, therefore, the original data that the model has not seen in its learning or training phase.

The suggested model’s solidity was assessed using the ten-fold cross validation technique [42]. First, using stratified random sampling, the EEG signals are randomly divided into 10 equal parts while maintaining the class label distribution for each fold. The model is trained using 80% of the EEG segments from each fold, with the remaining 20% being utilized to evaluate the performance of the suggested model. To monitor the training process and avoid the model over-fitting, we further divided the training data into actual training data (90%) and validation data (10%). The procedure is repeated ten times, with each iteration training a fresh model with fresh training and testing data. The validation set’s classification outcomes are used to optimize the model after it has been trained using the training set.

In this study, four evaluation measures were used: accuracy, precision, sensitivity, and specificity.

- The proportion of correctly identified instances to the total number of instances is represented by the accuracy (*Acc*). For the multi-class problem, *Acc* was calculated as follows:
  $$\begin{array}{c} Acc=\frac{1}{N}\sum_{\left(C=1\right)}^{N}\frac{\left(T_{P}^{C}+T_{N}^{C}\right)}{\left(T_{P}^{C}+T_{N}^{C}+F_{P}^{C}+F_{N}^{C}\right)} \end{array}$$ (5)
  Where *T*_*P*_ stands for true positives, *F*_*P*_ stands for false positives, *T*_*N*_ represents true negatives, *F*_*N*_ stands for false negatives, *C*depicts the class index, and *N* specifies the total number of classes.
- Error rate or classification error shows the percentage of predictions that were incorrect. It is calculated by (*F*_*P*_ + *F*_*N*_)/(*T*_*P*_ + *T*_*N*_ + *F*_*P*_ + *F*_*N*_).
- The precision (*Prec*) or positive predictive value (PPV) shows about the fraction of predictions as a positive class were in actual positive. *Prec* is calculated as:
  $$\begin{array}{c} Prec=\frac{1}{N}\sum_{\left(C=1\right)}^{N}\frac{\left(T_{P}^{C}\right)}{\left(T_{P}^{C}+F_{P}^{C}\right)} \end{array}$$ (6)
- Sensitivity (Sen) or Recall is also called as True Positive Rate (TPR) or Probability of Detection. Sen basically provides the percentage of all positive samples that the classifier has accurately predicted as positive. Sen is estimated as follows:
  $$\begin{array}{c} Sen=\frac{1}{N}\sum_{\left(C=1\right)}^{N}\frac{\left(T_{P}^{C}\right)}{\left(T_{P}^{C}+F_{N}^{C}\right)} \end{array}$$ (7)
- Specificity (Sp) is also called as True Negative Rate (TNR) or selectivity. Sp identifies the percentage of all negative samples that the classifier has accurately predicted as negative. Sp is estimated as follows:
  $$\begin{array}{c} Sp=\frac{1}{N}\sum_{\left(C=1\right)}^{N}\frac{\left(T_{N}^{C}\right)}{\left(T_{N}^{C}+F_{P}^{C}\right)} \end{array}$$ (8)
- False Positive Rate (FPR), or Type I Error is the samples incorrectly predicted as positive out of total actual negatives. FPR is calculated by:
  $$\begin{array}{c} FPR=\frac{F_{P}}{F_{P}+T_{N}} \end{array}$$ (9)
- False Negative Rate (FNR), or Type II Error is the samples incorrectly predicted as negative out of total actual positives. FNR is calculated by:
  $$\begin{array}{c} FNR=\frac{F_{N}}{F_{N}+T_{P}} \end{array}$$ (10)
- The F1 score was calculated using the precision (*Prec*) and (*Sen*) as,
  $$\begin{array}{c} F1-Score=2\times (\frac{\left(Prec\times Sen\right)}{\left(Prec+Sen\right)}) \end{array}$$ (11)
- The kappa coefficient (*K*) measures the agreement between predicted and true values. The higher is the *K* value, the better the performance of the classifier, i.e, *K* = 1 represents perfect agreement and *K* = 0 represents no agreement. *K* is computed as follows:
  $$\begin{array}{c} K=\frac{c\times s-\sum_{n}^{N}p_{n}\times t_{n}}{s^{2}-\sum_{n}^{N}p_{n}\times t_{n}} \end{array}$$ (12)
  where *c* is the total number of elements that are correctly predicted, *s* is the total number of elements, *p*_*n*_ denotes the number of times that class *n* was predicted (the sum of column *n*), *t*_*n*_ is the number of times that class *n* truly occurs (the sum of row *n*), and *N* is the total number of classes.

### Results

The ResNet model is build using Keras and Tensorflow GPU backend. After training process of the model, the network parameters were saved in the HDF5. The learning rate and the batch size have a key role in achieving the best accuracy in the automatic ECG classification. The suggested model was tested in a variety of experiments with varied learning parameter values. The speed of convergence was quite slow for a smaller value of the learning rate (i.e., less than 0.0005). The larger values also lead to low convergence. After several tests, the learning rate was set to be 0.001 and the Adam optimizer is used.

**Fig 6:**
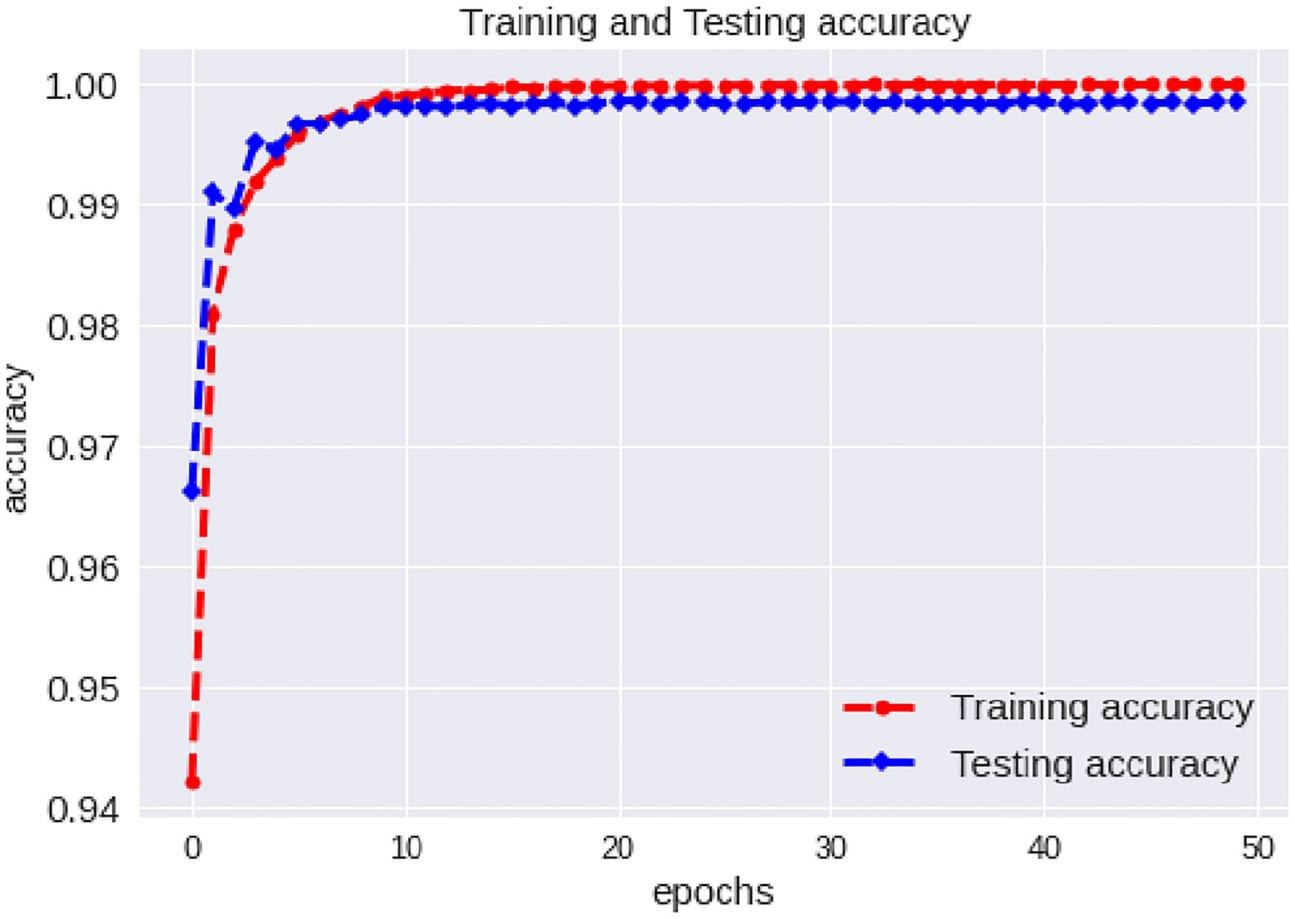
Training and testing accuracy (batch size = 32).

**Fig 7:**
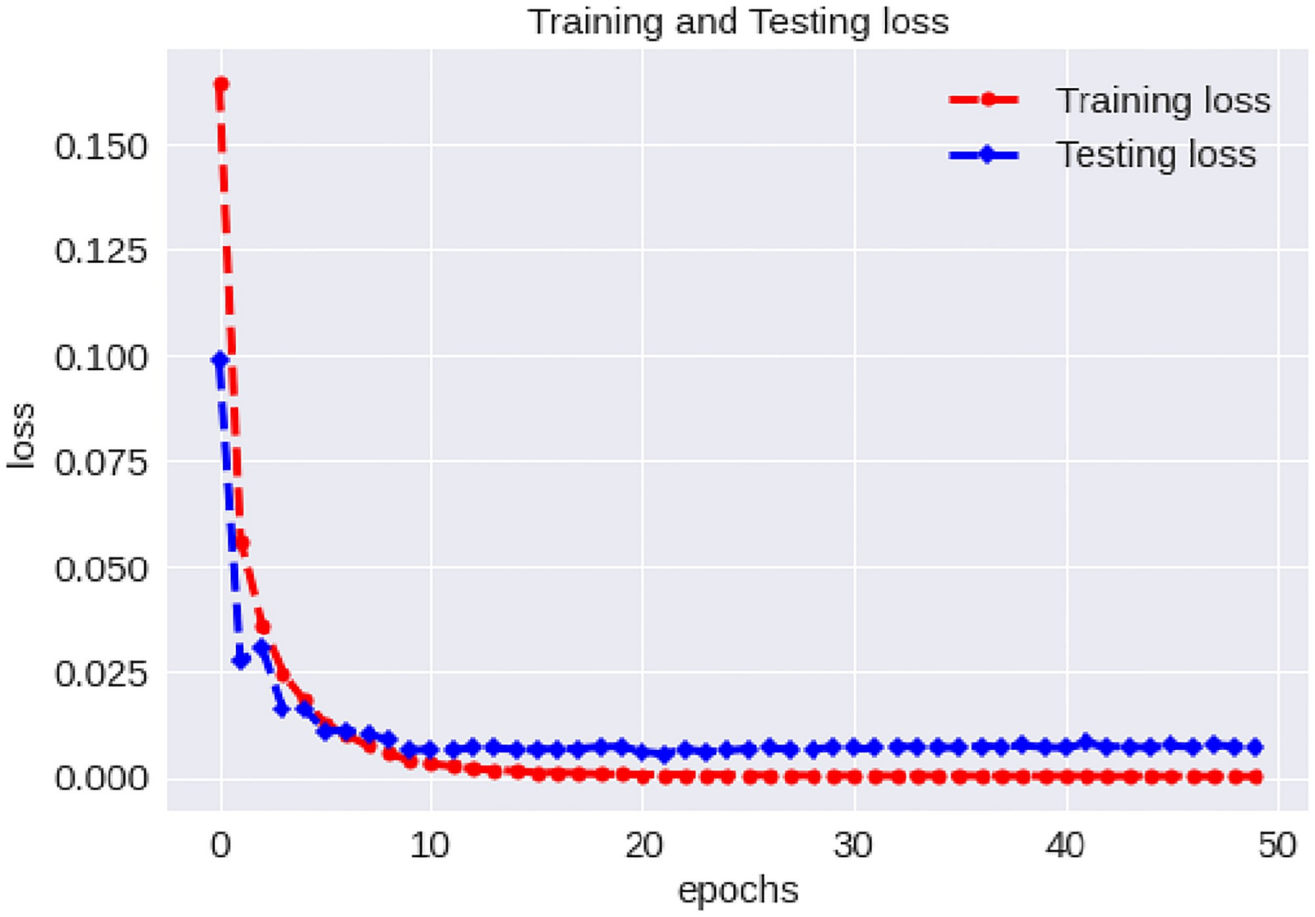
Training and testing loss.

**Fig 8:**
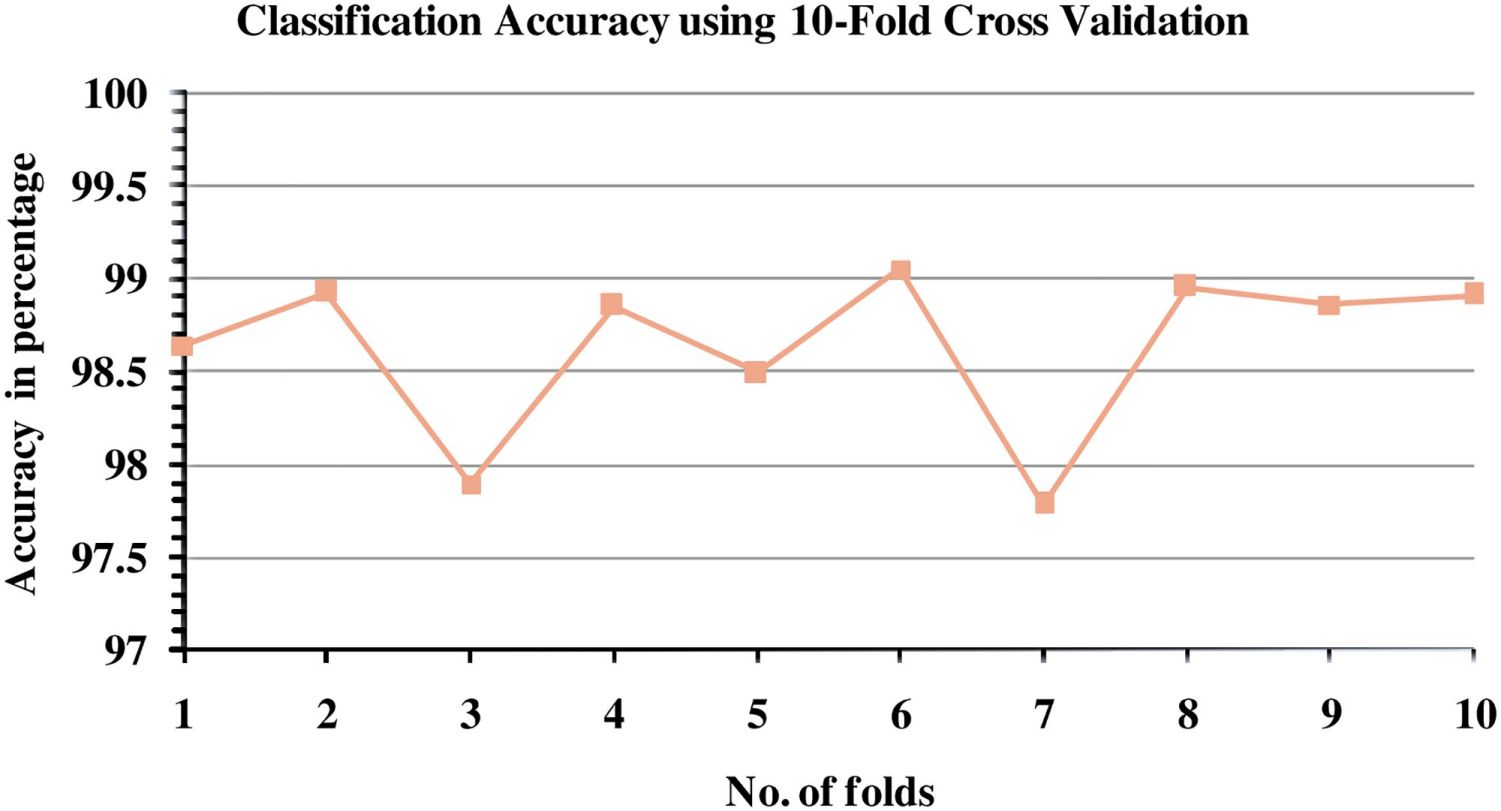
Accuracy using 10-Fold cross validation.

**Fig 9:**
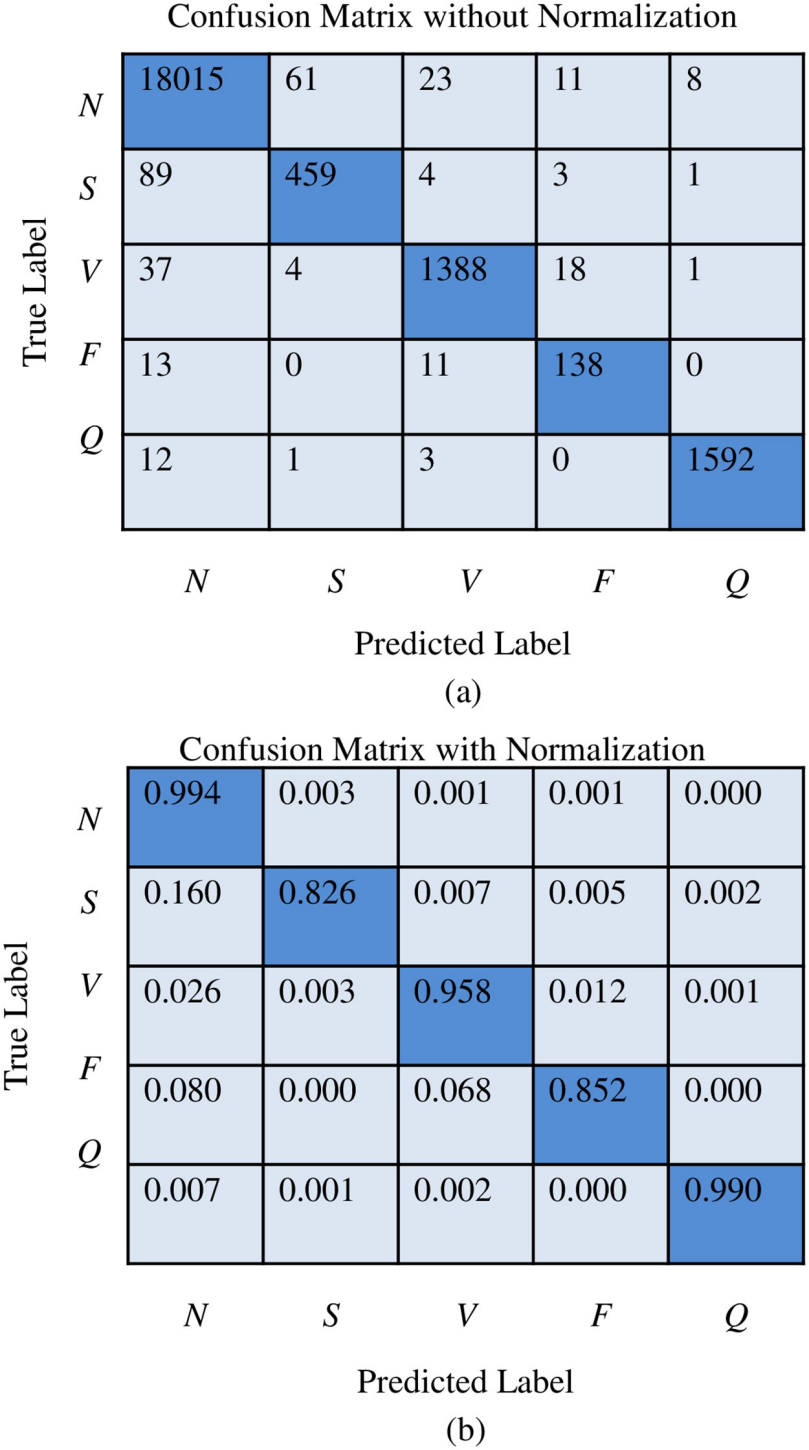
Classifier performance using confusion matrix (a) Without normalization (b) With normalization.

**Fig 10:**
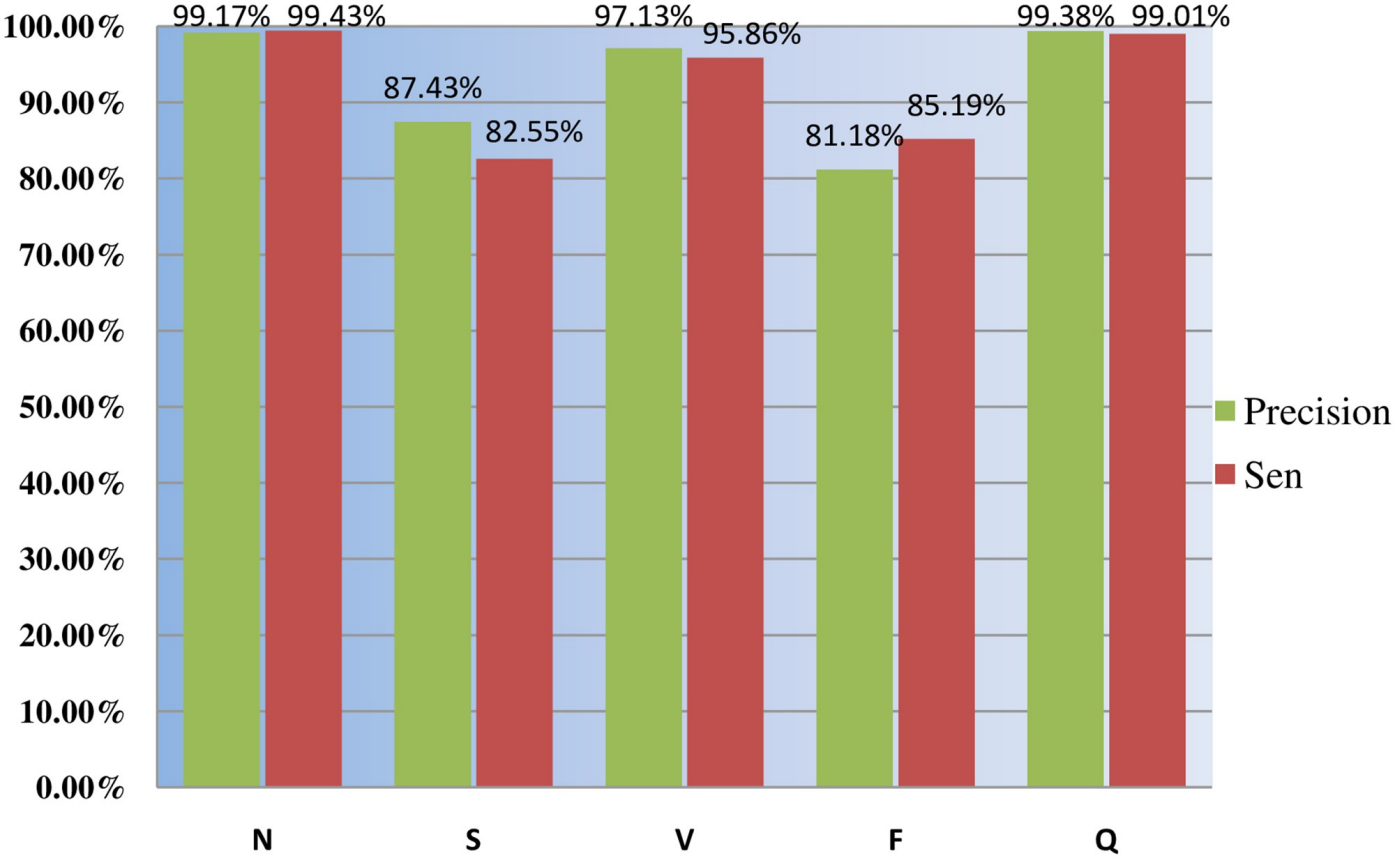
Precision and sensitivity values for five classes.

By selecting the batch size to be 32 and epochs equal to 50, the plots for training and testing accuracy were obtained as shown in Fig 6. It is evident from Fig 6 that training and testing accuracy increases with epochs. Initially, there were few valleys in the testing accuracy but after 9th epoch both curves become converging and reaches a stable state. Fig 7 shows training and testing loss curves. Initially, the testing loss shows abrupt characteristics but after 9th epoch the testing loss becomes steady with no abnormal fluctuations. 10-fold cross validation is employed to evaluate the model performance. The fold-wise accuracy plot is shown in Fig 8 providing the highest accuracy of 99.05% and average accuracy of 98.62% for the ten-folds. Without using SMOTE, the average accuracy achieved is 95%, which is far low than the accuracy obtained by employing SMOTE. Fig 9 provides the performance of proposed model using confusion matrices for all five classes without and with normalization. The diagonal elements reflect successfully categorized classes, while anything off the diagonal indicates improper categorization. The averaging of the diagonal values in the normalized confusion matrix provides the average accuracy of the classification system. Using 10-fold CV on ECG test dataset, Table 3 provides average values of different metrics such as precision, recall, F1-score, and *K*. *Prec* and *Sen* values for five classes are depicted in Fig 10. The 10-fold cross validation provides an average *Prec* and *Sen* values of 92.86% and 92.41% respectively, while the average *K* value is 95.5% (0.955). In DL, there are several challenges involved in the robust architecture of CNN. Among them, the appropriate hyper-parameters adjustment is a crucial as it will have an impact on the network’s performance as it approaches convergence. One of the most important hyperparameters to consider is the batch size. The batch size is the number of ECG data that will be utilized in the gradient estimation process, and it is one of the most important hyper-parameters to tune before starting the training process. By setting different batch size, we have evaluated the impact of batch size on network performance in terms of overall accuracy and convergence. In general, a small batch can converge more quickly than a large batch, while a large batch can achieve an optimum minimum that is not possible with small batch.

**Table 3:** Statistical performance on ECG test dataset.

Class	Prec%	Sen%	Sp%	FPR(%) Type-I error	FNR(%) Type-II error	Error rate	F1-Score(%)	Accuracy(%)	K(%)
*N*	99.17	99.43	96.01	0.040	0.006	0.0116	99.38	-	-
*S*	87.43	82.55	99.69	0.003	0.174	0.0075	84.92	-	-
*V*	97.13	95.86	99.80	0.002	0.0414	0.0047	96.50	-	-
*F*	81.18	85.19	99.85	0.001	0.148	0.0026	83.14	-	-
*Q*	99.38	99.01	99.95	0.000	0.00996	0.0013	99.19	-	-
*Average*	92.86	92.41	99.06	0.009	0.076	0.0054	99.63	98.63	95.53

In this paper, the learning rate and the batch size are two important optimization factors that are used in the proposed model to evaluate the performance. These two op-timization parameters must be carefully chosen to get the best accuracy in the automatic categorization of arrhythmia using ECG signals in order to increase performance. The suggested model was evaluated with different learning rates and batch sizes using Adam optimizer.

Firstly, we used different learning rates with the batch sizes B = [32, 64, 100, 500, 1024, 2048, 3000, 4000, 6000, 8000, 10000] for fine-tuning the network. The number of epochs was set at 50 for consistency of results and due to the huge size of the ECG dataset. For leaning rates less than 0.0001, the speed of convergence is very slow. The stability and speed of convergence are improved as learning rate is increased.

When learning rate was set as 0.0001, Table 4 provides accuracy value for different batch sizes with fixed learning rate of 0.0001. The accuracy was highest and reached a stable state for a batch size of 1024. When leaning rate is set to 0.001, an optimum value of accuracy is obtained with stable state for a batch size of 32. Table 5 provides accuracy value for different batch sizes with fixed learning rate of 0.001. In this case, a general trend of decrease in accuracy is observed as batch size increases.

**Table 4:** Performance on ECG test dataset using different batch size for a learning rate of 0.0001.

Batch size	Accuracy
32	98.30
64	98.25
100	98.54
500	98.42
1024	98.63
2048	98.33
3000	98.11
4000	98.12
6000	97.84
8000	97.66
10000	97.62

**Table 5:** Performance on ECG test dataset using different batch size for a learning rate of 0.001.

Batch size	Accuracy
32	98.72
64	98.58
100	98.56
500	98.50
1024	98.34
2048	98.26
3000	98.15
4000	98.22
6000	97.71
8000	97.61
10000	96.93

## Discussion

Table 6 highlights the performance comparison works published in literature by numerous ECG classification algorithms employing ML and DL concepts. Using the MIT-BIH arrhythmia database, these approaches had the best overall classification performance. The existing models utilize different training and testing datasets and diverse CNN architectures with numerous classification classes. Therefore, it is not suitable to directly compare the proposed deep ResNet model with existing techniques. Numerous ECG classification approaches for the categorization of arrhythmia have employed 1-D approach to their models using approaches such as SVM, K-NN, LSTM and CNN. In [43] authors proposed a DL based ResNet-LSTM classier combined with genetic algorithm for optimal feature combination. Their model is computationally complex and provides an average classification accuracy of 98%. However, our proposed 1-D deep ResNet classier provides superior performance in terms of average accuracy.

**Table 6:** Comparison results with the state of the art.

Authors	Year	Proposed model/classifier	No. of classes	Accuracy(%)	Prec(%)	Sen(%)	Sp(%)
Kallas et al. [44]	2012	KPCA + SVM	3	97.17	-	-	-
Kumar and Kumaraswamy [45]	2012	RF		92.12	-	-	-
Martis et al. [46]	2013	Neural Network and SVM	5	93.48	99.33	99.27	98.31
Park et al. [47]	2013	K-NN	17	97.02	-	97.1	96.9
Lin et al. [48]	2014	Linear discriminant	4	93	-	-	-
Raj et al. [49]	2015	Rule based approach	4	97.96	96.46	97.72	99.09
Li et al. [13]	2016	RF, SVM, DT, PNN, K-NN	5	94.61	-	-	-
Acharya et al. [33]	2017	Deep CNN	5	94.03	97.86	96.71	91.54
Sahoo et al. [50]	2017	DWT, SVM	4	98.39	96.85	96.86	98.92
Yang et al. [51]	2018	Linear SVM	5	97.94	-	-	-
Kachuee et al. [52]	2018	Deep CNN	5	93.4	-	-	-
Oh et al. [42]	2018	LSTM and CNN	5	98.10	-	97.50	98.70
Rajkumar, A. et al. [53]	2019	1-D CNN	7	93.60	-	-	-
Izci, E. et al. [54]	2019	2-D CNN	5	97.42	-	-	-
Pandey SK, and Janghel RR. [55]	2019	11-layer CNN	5	98.3	86.06	95.51	-
Proposed	2023	Deep ResNet	5	98.63	92.86	92.41	99.06

## Conclusion

In this paper, 1-D convolutional ResNet model is proposed for the classification of five heartbeat types taken from PhysioNet MIT-BIH Arrhythmia database, which is freely available and widely utilized database for Arrhythmia classification. The entire data was divided into 80-20 train test split. We used SMOTE on training dataset only whereas testing dataset was not oversampled to preserve its originality. SMOTE creates data balancing where all the heartbeat classes have an equal number of samples in the training dataset. The training data is passed to the deep layer 1-D convolutional ResNet classifier. The model provides an average accuracy of 98.63% to classify different heartbeat signals. Therefore, the proposed model can provide effective diagnostic mechanism for heartbeat classification problems.

The procedures involved in finding the cardiac arrhythmias is a time-consuming process that requires a clinical professional to carefully observe recordings that can last for hours. CNN classifiers can enhance the performance of clinical specialists through these automated features learning CNN. This would help to enhance the clinical diagnosis and treatment of some of the most serious cardiovascular diseases.
